# The prevalence of hepatitis C and hepatitis B in lesbian, gay, bisexual and transgender populations: a systematic review and meta-analysis

**DOI:** 10.1186/s40001-022-00677-0

**Published:** 2022-03-26

**Authors:** Ghobad Moradi, Marzieh Soheili, Roya Rashti, Hojat Dehghanbanadaki, Elham Nouri, Farima Zakaryaei, Elnaz Ezzati Amini, Sheno Baiezeedi, Sanaz Ahmadi, Yousef Moradi

**Affiliations:** 1 Social Determinant of Health Research Center, Research Institute for Health Development, Kurdistan University of Medical Sciences, 6617713446 Sanandaj, Iran; 2 Faculty of Medicine, Kermanshah University of Medical Sciences, 6714415153 Kermanshah, Iran; 3 Endocrinology, and Metabolism Research Centre, Endocrinology and Metabolism Clinical Sciences Institute, Tehran University of Medical Sciences, 1416753955 Tehran, Iran; 4 Department of Emergency Medicine, Kurdistan University of Medical Sciences, 6617713446 Sanandaj, Iran; 5 Department of Epidemiology, School of Public Health, Iran University of Medical Sciences, 1449614535 Tehran, Iran; 6 Department of Epidemiology and Biostatistics, Faculty of Medicine, Kurdistan University of Medical Sciences, 6617713446 Sanandaj, Iran

**Keywords:** LGBT, Lesbian, Gay, Bisexual, Transgender, Hepatitis B, Hepatitis C, Meta-analysis

## Abstract

**Objectives:**

This study aimed to systematically review the literature on the prevalence of hepatitis C virus (HCV) and hepatitis B virus (HBV) infections in transgender and LGBT people and determine their pooled estimates worldwide.

**Methods:**

Databases (PubMed, Scopus, Web of Science, Embase, Ovid, Cochrane, PsycInfo) were searched from April 2000 to July 2021. The analyses were executed using the random-effects model in Stata 16.

**Results:**

Ten studies, including eight studies on four transgender people and two studies on 2150 LGBTs, were included. The pooled prevalence of HCV and HBV in all transgender populations globally were 9% (95% CI 3–15%) and 11% (95% CI 2–20%), respectively. The corresponding prevalence in male-to-female transgender people were estimated as 5% (95% CI 1–9%) and 6% (95% CI 3–10%), respectively. These estimates in American transgenders were 10% (95% CI 5–25%) and 16% (95% CI 8–23%), respectively.

**Conclusion:**

This study was identified the overall prevalence of HBV and HCV infections in transgender people, which were higher than those in the general population.

## Introduction

Transgender are people who are phenotypically born male at birth, but are known as women or vice versa. These people face numerous health, social and medical risks. These risks expose transgender people to various infections, especially sexually transmitted and blood-borne diseases (STBBIs) like hepatitis C, hepatitis B, and AIDS [1]. In addition, this population encountered many problems such as lack of proper access to health services, social stigma, discrimination, rejection by families and friends, mental and psychological issues, and many economic and social challenges that made them perform risky sexual non-sexual behaviors. Sex trade, sexual intercourse for money, having several partners, taking numerous therapeutic and illegal hormones, drugs, and alcohol consumption are among the most common high-risk behaviors associated with many common infections such as hepatitis B virus (HBV) and hepatitis C virus (HCV) infections in this group and sexual minorities [2, 3].

HBV is transmittable through injectable drugs and sexual contact in these people. On the other hand, due to having different sexual partners, it would be possible for this infection to be developed in the transgender population if they had sex with individuals infected with HBV or other high-risk behaviors [4, 5]. Although this is not common, HCV is also transmittable through sexual activity. It seems that having STBBIs, having sex with multiple partners, and performing unprotected anal sex increased the risk of HCV infection in the transgender population [6, 7]. On the other hand, this group of sexual minorities is at risk of hepatitis B and C because of consuming different and illegal hormones and repeated injections. Of course, having risky behaviors such as substance abuse and drug injection also increases the risk [8, 9]. Moreover, owing to economic problems, discrimination, and social stigma, they cannot provide sterile syringes for each injection of hormones per week or month. So, they use shared needles to inject hormones, which puts them at higher risk for viral hepatitis, especially hepatitis C.

Therefore, determining the prevalence of HCV and HBV in transgender people and other sexual minorities could be important for health policymakers and health workers to develop a prevention and treatment protocol related to HCV and HBV, such as hepatitis B vaccination or hepatitis C treatment or screening programs. Achieving the 2030 goals of controlling viral hepatitis required attention to high-risk groups [10–12]. Few studies have been conducted to determine the prevalence of hepatitis C and B in transgender people, but the obtained values are controversial. They could not be instrumental in developing health programs and treatment facilities. This study aimed to investigate the prevalence of HCV and HBV in transgender people and LGBT populations worldwide using a systematic review and meta-analysis approach.

## Methods, search terms, and search strategies

The present study was a systematic review and meta-analysis designed and performed based on PRISMA and MOOSE criteria [13, 14]. The search was conducted from April 2000 to July 2021 in international databases involving PubMed (Medline), Scopus, Web of Sciences, Embase, and Ovid. In addition, for gray literature, the correlated databases with HBV/HCV and LGBTs such as UNAIDS, UNDP, WHO, CDC were also searched. The related reports would be analyzed if they were not paradoxical with inclusion criteria. The keywords including HBV/HCV, Lesbian, Gay, Bisexual, LGBT, and Transgender are used to develop a search strategy. These keywords were found using Mesh and Emtree, and then the search strategy was designed and carried out following each international database.

## Eligibility criteria

Inclusion criteria involved cross-sectional or analytical cross-sectional or retrospective studies. The studies which aimed to determine the prevalence or frequency of HBV/HCV in LGBT people as their primary outcomes were also involved. Only studies in English were evaluated for analysis. Non-English studies, cohorts, case–control studies, clinical trials, letters to the editor, and systematic reviews were withdrawn from this meta-analysis. In addition, studies conducted by the statistical population other than LGBT people were also excluded from the study.

## Selection and screening

The results were entered into Endnote software version 8. After removing the repeated cases, screening articles based on titles, abstracts, and full-texts were performed considering inclusion and exclusion criteria. Ultimately, after the final selection of articles, a manual search was also performed, and the relating items and purpose of the study were added to the last reports for analysis.

## Data extraction

A meeting was held with the experts in HBV/HCV infections and high-risk groups such as LGBT epidemiologists and infectious disease specialists to extract the data. A checklist for data extraction was developed and designed. Then, data were extracted using this checklist from related articles. The item in this checklist included the author's names, years of publication, countries, sample sizes, age, types of studied populations, HBV, and HCV detection methods.

## Risk of bias

Two authors evaluated studies based on the Newcastle Ottawa Scale (NOS) Quality Assessment checklist [15]. This checklist was designed for qualitative evaluation of observational studies, predominantly cross-sectional and retrospective ones. This tool examined each study by six items in three groups, including selecting study samples, comparing and analyzing the study groups, and measuring and analyzing the desired outcome. The maximum score for each study was 9 points.

Each of these items would have been given a score of 1 if they were observed in the studies. In case of a difference in the score allocated to the published articles and reaching the agreement, the discussion method and the third researcher were applied.

## Statistical analysis

First, the prevalence values were extracted from the selected studies to perform this meta-analysis. Then the standard error of the prevalence values for each of the studies was calculated. In addition to the prevalence, the number of patients with HBV/HCV was extracted from the total sample size in each study. In this research, the DerSimonian–Laird random-effects model was used to estimate the cumulative prevalence of HBV/HCV with a 95% confidence interval (95% CI) in transgender people using Metaprop and Metan commands in Stata 16.

Cochran *Q* and *I*^2^ tests were also used to investigate the heterogeneity and variance between the selected studies for meta-analysis. According to the Cochrane criteria and *I*^2^ index, the amount of heterogeneity was divided into four categories: 0–40% (may not be necessary), 30–60% (may represent moderate heterogeneity), 50–90% (may represent significant heterogeneity), and finally 75% and beyond (considerable heterogeneity) [16, 17].

Funnel plot and Egger tests were used for evaluating the publication bias. Also, the meta-regression diagram and analysis were used to investigate the relationship of age variables of transgender people and the sample size of selected studies with estimated cumulative prevalence. The cumulative meta-analysis was also performed based on the publication date of the included studies. All bilateral statistical tests were considered with *α* = 0.05.

## Results

**Fig. 1:**
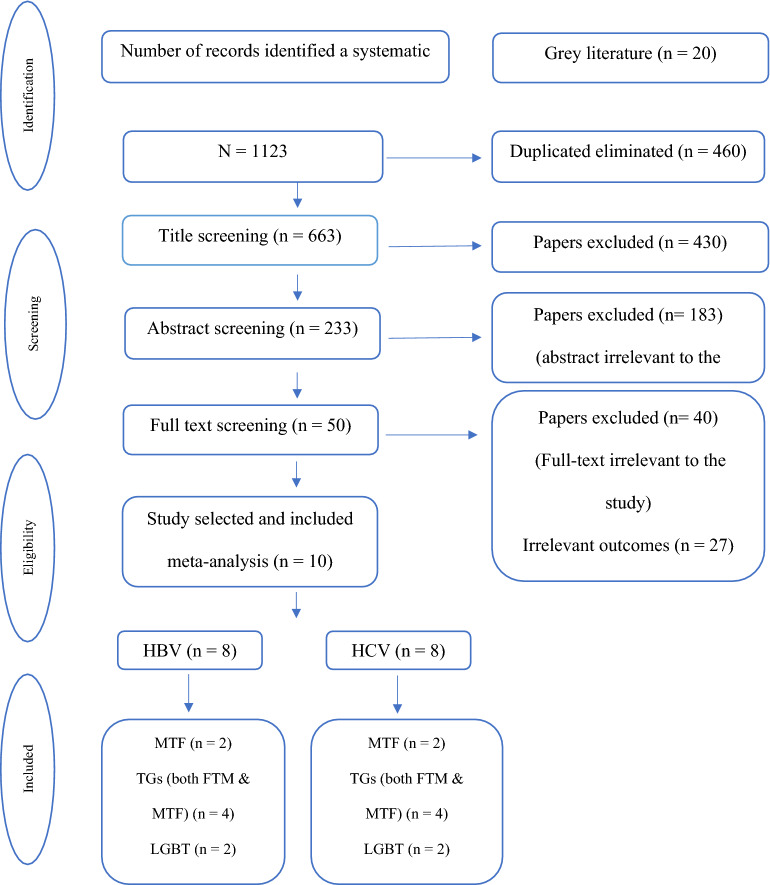
The search outputs and study selection

At the initial search strategy, 1123 articles were found. First, the duplicates were removed, and then 663 articles were entered into the screening stage according to the title. Four hundred thirty articles were removed at this step, and 233 papers were evaluated based on their abstracts. Then, 50 articles remained and entered into the screening phase based on their full-texts. Of these articles, 27 were excluded due to the non-related outcomes to the present study, 11 cases because of different statistical populations, and 2 cases owing to lack of full-text availability. Finally, ten studies [2, 18–26] were entered into the analysis. Out of these articles, eight studies [2, 18–22, 24, 26] reported the prevalence of HBV, and eight studies [2, 18–20, 22, 23, 25, 26] reported HCV prevalence in transgender and LGBT people (Fig. 1).

Table 1 lists the general characteristics of the included studies. Of all studies, two [18, 19] had been conducted in the LGBT community (Total sample size = 2150), and eight [2, 20–26] in the transgender community (Total sample size = 4273). Seven studies were conducted with cross-sectional design in Argentina [2], The Republic of Dominican [18], Spain [19], the United States [24], Nigeria [25], and Indonesia [26]; in contrast, three ones had been retrospectively conducted in Pakistan [21], Italy [20], and the United States [23].

**Table 1:** Baseline characteristics of the included studies

Authors (year)	Type of study (country)	Study population (type)	Age (mean)	Sample size (*N*)	HBV prevalence *N* (%)	HCV prevalence *N* (%)	Number (type of laboratory test)
Johnston et al. [16]	Cross-sectional (Dominican Republic)	LGBT	≥ 15	1388	28 (2.05%)	53 (3.85%)	HBV: HBsAg BIO-CARD HCV: Bioblot HCV
Gutierrez et al. [17]	Cross-sectional (Spain)	LGBT	18–41	762	27 (3.5%)	6 (0.8%)	HBV: microparticle Enzyme Immunoassay HCV: ELISA, line immunoassay
Carobene et al. [2]	Cross-sectional (Argentina)	TGs (Both)	29	273	106 (40%)	12 (4.5%)	ELISA, particle Agglutination
Luzzati et al. [18]	Retrospective (Italy)	TGs (Both)	33.5 ± 7.7	243 (MTF: 218 FTM: 25)	MTF: 10 (4.6%) FTM: 2 (4%)	MTF: 8 (3.5%) FTM: 2 (8%)	HBV: immunoassays Enzygnost, Chemiluminescent microparticle Immunoassay, CMIA HCV: immunoassay Ortho HCV 3.0 Elisa Test System, immunoblotting Chiron Riba HCV 3.0 SIA
Ahsan et al. [19]	Retrospective (Pakistan)	TGs (both)	All ages	877	NR	128 (14.6%)	HCV: RAPID ICT test, ELISA
Brito et al. [20]	Cross-sectional (Dominican Republic)	MTF	≥ 18	100	4 (4%)	1 (1%)	HBV: hepatitis B surface antigen (HBsAg II, COBAS, Roche Diagnostics) HCV: hepatitis C antibody (anti-HCV II, COBAS, Roche Diagnostics)
Krieger et al. [21]	Retrospective (USA)	TGs (Both)	35.5–41.3	1005	48 (4.8%)	NR	HBV serology (surface antigen and antibody)
Facente et al. [22]	Cross-sectional (USA)	MTF	> 30	951	NR	236 (24.8%)	HCV: ELISA
Adeyemi et al. [23]	Cross-sectional (Nigeria)	MTF	25	717	69 (10%)	NR	HBV serology (surface antigen and antibody)
Hadikusumo et al. [24]	Cross-sectional (Indonesia)	TGs (Both)	19–60	107	10 (9.3%)	7 (6.5%)	HBV: reverse passive hemagglutination assay, PCR HCV: reverse particle hemagglutination method, PCR

## Prevalence of HCV and HBV in LGBT population

Among the included studies, Johnston et al. [18] and Gutierrez et al. [19] reported the prevalence of HCV and HBV in the LGBT population, i.e., lesbian, gay, bisexual, transgender altogether. Johnston et al. [18] estimated the prevalence of HCV as 3.85% and HBV as 2.05% among the LGBT population of the Dominican Republic, and Gutierrez et al. [19] stated the prevalence of HCV and HBV were 0.8% and 3.5% among the LGBT people of Spain, respectively. We did not pool the results of these studies with other included studies since their target population included all lesbians, gays, bisexuals, and transgender people while other included studies investigated only the transgender community. Besides, we will update our study for pooled analysis of these corresponding estimates when further studies have been conducted on the LGBT population.

## Prevalence of HCV in the transgender population

**Fig. 2:**
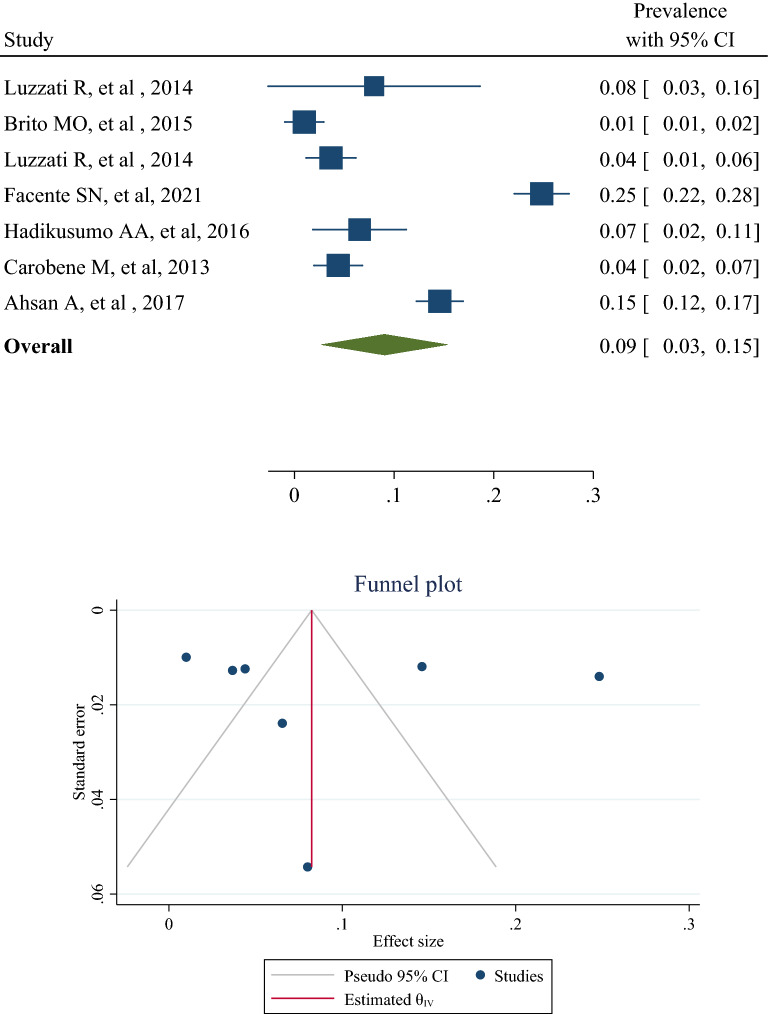
The forest plot and publication bias of hepatitis C prevalence in transgender populations

The results of 6 studies [2, 20–22, 24, 26] reporting the prevalence of HCV in transgender people were combined and showed that the overall prevalence of HCV in the transgender population around the world was 9% (95% CI 3–15%) (Fig. 2). The heterogeneity percentage of this estimate was 77.16% (*p* < 0.01). The lowest and highest prevalence among these studies was 1% in Brito et al. study [22] and 25% in Facente et al. study [24], respectively. There are hints for publication bias (Egger’s test, *p* = 0.043). The meta-regression showed that the variables of sample sizes and age of individuals had no significant effect on the cumulative prevalence of HCV in transgender people (Table 2).

**Table 2:** Meta-regression on the heterogeneity of pooled prevalence

	Variables	Coefficient	Standard error	*p* value	95% CI	
					Lower	Upper
HCV in TGs	Age	− 0.122	0.043	0.655	− 0.200	0.021
	Sample size	0.002	0.001	0.412	− 0.002	0.004
HBV in TGs	Age	− 0.091	0.072	0.968	− 0.117	0.078
	Sample size	0.005	0.001	0.751	− 0.004	0.007

## Prevalence of HBV in the transgender population

**Fig. 3:**
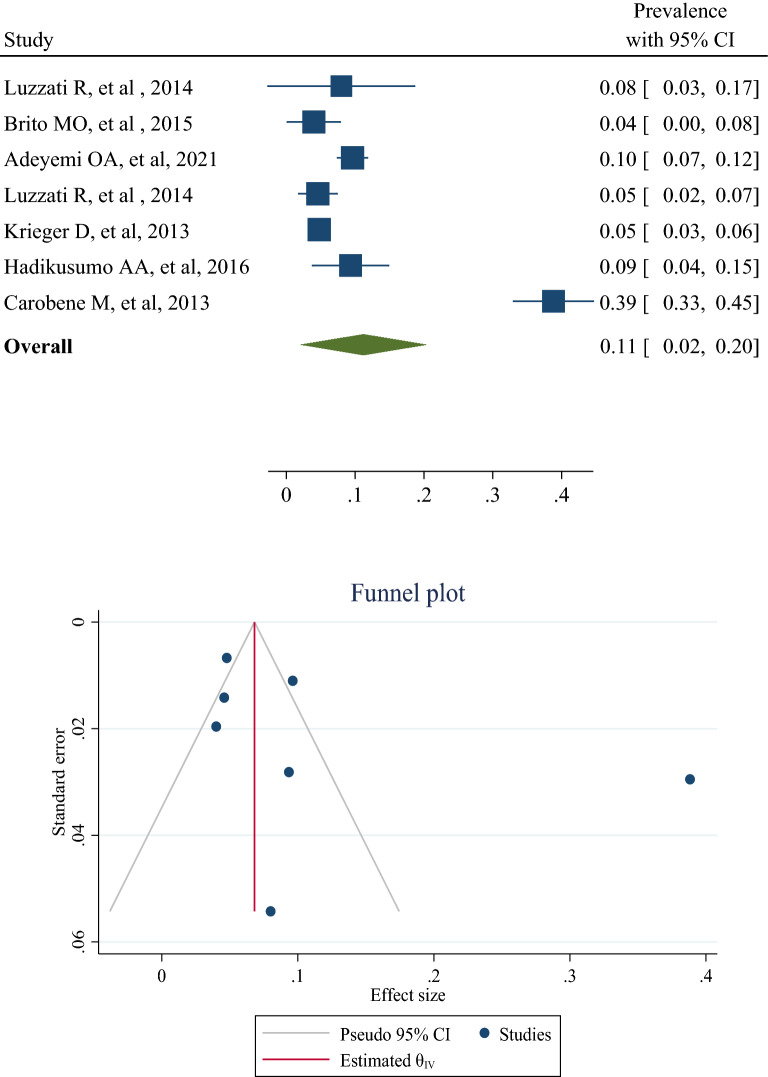
The forest plot and publication bias of hepatitis B prevalence in transgender populations

Six studies [2, 20, 22, 23, 25, 26] reported the prevalence of HBV in transgender people. Luzzati et al. [20] reported this prevalence in male to female and female to male, separately. Therefore, seven prevalence records of HBV in transgender people were combined and showed that the overall prevalence of HBV in transgender people in the world was 11% (95% CI 2–20%). Heterogeneity was 91.43%, which was substantially high (Fig. 2). The lowest and highest prevalence was 4% in Brito et al. study [22] and 83% in the Carobene et al. study [2] (Figs. 2 and 3). There are hints for publication bias (Egger’s test, *p* = 0.019). The results of meta-regression are also expressed in Table 2. Age and sample sizes had no significant effect on the pooled prevalence of HBV in transgender people (Table 2).

## Cumulative meta-analysis

**Fig. 4:**
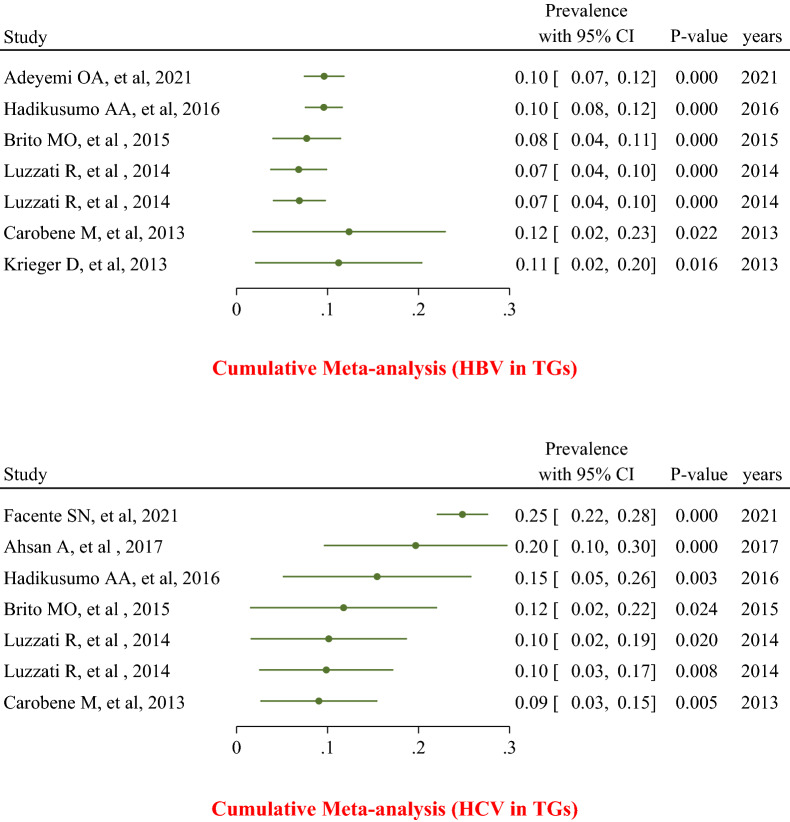
The cumulative meta-analysis of the pooled prevalence of hepatitis C and B

The cumulative meta-analysis was performed to determine the impact of adding each study on the pooled cumulative prevalence in the order of their publication date. For most HCV in transgender people, the cumulative meta-analysis reported a higher prevalence of HCV in recent studies (20%; 95% CI 10–30%) compared to the final incremental majority (9%; 95% CI 3–15%; Fig. 4a). Meanwhile, the cumulative meta-analysis concerning the prevalence of HBV in transgender people revealed that the recent reports on HBV prevalence in this population (10%; 95% CI 8–12%) were almost the same as the final cumulative prevalence (11%; 95% CI 2–20%; Fig. 4b).

## Subgroup analysis

The results of subgroup analysis based on the type of gender reassignment (male to female or female to male) and continent are shown in Table 3. Three studies [20, 22, 24] reported HCV prevalence in transgender people who had changed gender from male to female. After combining these results, the prevalence of HCV in male-to-female transgender was estimated as 5% (95% CI 1–9%). The remarkable point was that the heterogeneity was 55.93%, much lower than the heterogeneity rate of the overall prevalence in Fig. 2. Therefore, it could be said that the failure to determine the type of gender change (from male to female and vice versa) in preliminary studies was a source of heterogeneity in the pooled analysis of the overall prevalence of HCV. Besides, three studies [2, 21, 26] did not specify the type of gender reassignment, and combining these studies demonstrated that the prevalence of HCV in this group was 9% (95% CI 2–15%); however, due to the lack of separation of transgender based on their gender reassignment, its heterogeneity rate was high (*I*^2^ = 90.38%; Table 3). The subgroup analysis based on the continent showed that the pooled prevalence of HCV in the transgender population was 11% (95% CI 3–19%) in Asia, 10% (95% CI 5–25%) in America, and 4% (95% CI 1–6%) in Europe.

**Table 3:** Subgroup analysis of the prevalence of HBV and HCV in TGs based on male to female, female to male, and continents

Population	Subgroup		Number of studies	Pooled prevalence (95% CI)	Between studies			Between subgroups	
					*I*^2^ (%)	*P* heterogeneity	*Q*	*Q*	*P* heterogeneity
HCV in TGs	Type	MTF	3	5% (1–9%)	55.93	0.90	15.99	3.57	0.17
		FTM	1	8% (1–14%)	–	–	–		
		Both	3	9% (2–15%)	90.38	0.00	36.68		
	Continent	Asia	2	11% (3–19%)	71.23	0.06	9.09	3.41	0.18
		America	3	10% (5–25%)	76.55	0.00	44.50		
		Europe	2	4% (1–6%)	0.00	0.44	0.60		
HBV in TGs	Type	MTF	3	6% (3–10%)	79.54	0.07	10.90	1.14	0.56
		FTM	1	8% (1–14%)	–	–	–		
		Both	3	18% (3–34%)	88.90	0.00	17.68		
	Continent	Asia	1	9% (4–15%)	–	–	–	8.27	0.04
		America	3	16% (8–23%)	76.55	0.00	44.8		
		Europe	2	5% (2–7%)	0.00	0.44	0.37		
		Africa	1	10% (7–12%)	–	–	–		

The subgroup analysis on HBV prevalence based on the type of gender reassignment showed that the prevalence of HBV in male-to-female transgender was 6% (95% CI 3–10%) while after combining the results of studies that had not separated the type of gender reassignment, the prevalence of HBV was 18% (95% CI 3–34%). However, the heterogeneity rate in these estimates were high (*I*^2^ = 79.54% and *I*^2^ = 88.90%, respectively). Besides, this analysis revealed that the pooled prevalence of HBV in transgender people living in America was 16% (95% CI 8–23%), and those living in Europe had the pooled HBV prevalence of 5% (95% CI 2–7%) (Table 3). It is noteworthy to declare that we could not generalize our findings of subgroup analysis on continents due to a small number of reports in each subgroup; thus, we required more data on each continent for accurate estimation of HCV and HBV prevalence among the transgender population living in each continent.

## Discussion

The primary purpose of this study was to systematically review the prevalence of HBV and HCV in transgender people and the LGBT population around the world and determine their pooled estimates.

In this study, the prevalence of HBV in transgender people was higher than that of HCV. The previous studies also stated this finding [27, 28]. According to the results of studies performed in the world, about 2/3 of male-to-female transgender people and ¾ of female to male transgender individuals had no immunity against HBV infection, which could be a severe warning to developed and undeveloped countries because, in these countries, the routine vaccination program of HBV had been performed in the general population for a long time [29, 30]. Another more critical point inferred from the previous studies was the referral for vaccination after the first round of hepatitis B vaccination in high-risk groups, especially transgender people. A study showed that only 30% of high-risk groups had been referred for the second round of hepatitis B vaccination. In Argentina, hepatitis B vaccination has been available since 1982. Even in many countries, the vaccination started in 2000, but contrary to the expectation, the program for this vaccination in high-risk groups had not been formulated and implemented [29–32].

In the present research, the prevalence of HCV in male-to-female transgender people was 5%. The results of a study conducted in the general population of Western Europe and Latin America demonstrated that the prevalence of HCV in the general population was 1.5–3.5%. The results of this meta-analysis to determine the prevalence of hepatitis C in LGBT people were consistent with the results of previous studies in Latin America and Western Europe. At the same time, in the male to the female transgender community, this amount was 8%. The overlap of various factors had caused the prevalence in these people to be higher than that in the general population. This population was exposed to risky behaviors such as unprotected sex and oral or injectable substances. Drug use and sexual relationships, and standard syringes increased the chance of developing HCV.

On the other hand, other STDs such as HIV in these people raised the possibility of infection exacerbation and HCV association [33–36]. This group of transgender people (male to female) needed to work or have a suitable job to cover their living expenses and the hormones required to undergo the gender reassignment process [36, 37]. The prevalence of infectious diseases such as HCV, HBV, and HIV in this population was high due to the weakness of their families and social support systems and engaging in risky behaviors, and inadequate access to health care services [38, 39]. On the other hand, unprotected sex was prevalent in male-to-female transgender people with their sexual partners, increasing HIV, HBV, and HCV infections. It was necessary to consider the protocol for providing preventive and therapeutic services to identify, prevent, and treat HCV and HBV diseases. In addition to HIV tests, they need to be screened for HBV and HCV infections. This screening could be essential in the transgender community because people with HCV are asymptomatic and could pass on the disease to those around them and their sexual partners. By identifying these people, higher prevalence and transmission of this infection could be prevented with other transgender people or their other sexual partners, including lesbian, gay, bisexual, and other sexual minorities [20, 40–42]. This study showed that the prevalence of HCV in Asian and American transgender people was higher than that of Europeans. Transgender people living in different Asian regions and countries, especially Southeast Asia, were exposed to high-risk behaviors. So, the risk and chance of developing viral infections such as HCV in these people would be higher than transgender people in European countries. Still, the significant point had access to receive educational, counseling, and therapeutic services related to viral diseases and infections such as HCV for these people. The transgender population living in European and American countries had more accessible access to HCV-related services. In Asian countries, these educational, counseling, and treatment services were not readily available to transgender people because of more significant stigma and discrimination. In a survey conducted in Asian countries involving Bangladesh, India, Indonesia, Maldives, Myanmar, Nepal, Sri Lanka, Thailand, and Timor-Leste, the results expressed that although certain services were provided for transgender people and other sexual minorities, the majority of people did not have proper access to them [43, 44]. In addition to lack of access to services, different and numerous sexes and unprotected sex were other causes that played an essential role in the high prevalence of this infection in the Asian transgender population.

The sources of heterogeneity in estimating the overall prevalence were also investigated in this research. The results exhibited that lack of gender reassignment (male to female and female to male) was one of the principal sources of high heterogeneity since in selected preliminary studies for further meta-analysis (5 out of 8 studies), these variables were not considered. On the other hand, the meta-regression results showed that the age of transgender people and the sample size in the initial studies had little effect on the heterogeneity of the cumulative prevalence of HCV and HBV. Therefore, this difference in age and sample sizes of preliminary studies entered into a meta-analysis was not the source of heterogeneity in this research.

To our knowledge, this study was the first meta-analysis in the world, determining the cumulative and overall prevalence of HBV and HCV in transgender and LGBT people worldwide. Determining this prevalence in these and other sexual minorities could help develop a program to provide services for them. However, the lack of a sufficient number of preliminary studies to conduct different subgroups based on female to male transgender, the type of hormones (oral or injectable), the existence of other infections such as HPV or HIV, and the kind of sampling were among the limitations of this research. Besides, further studies were needed as cross-sectional research and population-based cohort with high sample sizes to ascertain the exact prevalence of these infections in these sexual minorities. Also, due to a small number of studies conducted in each continent, the subgroup analysis based on the continent had low power in terms of generalizability. Of course, over the next few years, the authors will be able to update the results of this meta-analysis by increasing the studies on this issue.

## Conclusion

This meta-analysis identified the overall prevalence of HBV and HCV infections in transgender people worldwide and presented that these estimates were higher than those in the general population. This high rate of infections among these people was worrying. Healthy and humanitarian policies are necessary to improve people living and health conditions to achieve world health organization goals by 2030 to reduce hepatitis B and C. The current prevalence of these infections would rise in the absence of comprehensive, rapid, targeted, and acceptable prevention and treatment services. So, appropriate care is necessary to evaluate barriers to access to care and active education on the transmission of blood-transmitted viruses in these people. Eliminating viral hepatitis as a public health threat by 2030 would be achievable if viral hepatitis testing and treatment prevention services were available to all individuals at-risk. Screening of HBV, HCV, and starting treatment in these people, such as specific services and programs, vaccination, education, prevention, and treatment programs related to HCV and HBV, is necessary to cut the transmission chain of these infections in these populations and their sexual partners.

## Data Availability

The datasets generated and analyzed during the current study are not publicly available due to their sensitive and potentially personally identifiable nature. Still, they are available from the corresponding author on reasonable request.

## Abbreviations

LGBT: Lesbian, gay, bisexual, and transgender
HBV: Hepatitis B virus
PRISMA: Preferred Reporting Items for Systematic Reviews and Meta-Analyses
MOOSE: Meta-analyses of observational studies in epidemiology
HCV: Hepatitis C virus
STBBIs: Blood-borne diseases
CI: Confidence interval
UNAIDS: Joint United Nations Program on HIV/AIDS
UNDP: United Nations Development Programme
WHO: World Health Organization
CDC: Centers for Disease Control and Prevention
NOS: Newcastle Ottawa Scale
STD: Sex transmitted disease
